# 57884 Fast strain-encoded cardiac magnetic resonance detects immune checkpoint inhibitor associated cardiotoxicity

**DOI:** 10.1017/cts.2021.761

**Published:** 2021-03-30

**Authors:** Jason Cuomo, Elio Ragheb, Attila Feher, Jennifer M. Kwan, Steffen Huber, Hamid Mojibian, Dana C. Peters, Albert Sinusas, Lauren A. Baldassarre

**Affiliations:** Yale School of Medicine

## Abstract

ABSTRACT IMPACT: Advanced cardiac magnetic resonance imaging techniques can help to protect cancer patients from cardiotoxicity from immunotherapy with a more sensitive assessment of cardiac function with strain imaging for detection of abnormal cardiac function in the setting of normal left ventricular ejection fraction. OBJECTIVES/GOALS: Immune checkpoint inhibitors (ICI) are associated with fatal cardiotoxicity. Cardiac magnetic resonance (CMR) imaging can assess ICI-associated cardiotoxicity, but the utility of CMR strain imaging is unknown. We present a study of patients with ICI-associated cardiotoxicity evaluated with fast strain-encoded (fast-SENC) CMR. METHODS/STUDY POPULATION: This prospective study was approved by the institutional IRB and informed consent was obtained from 15 patients (5 patients with ICI-associated cardiotoxicity, 10 controls patients) between August 2018 and January 2020. All patients with ICI-associated cardiotoxicity had abnormal troponin values and evidence of cardiotoxicity on T2-weighted and/or delayed enhancement CMR images. All patients underwent standard CMR assessment with steady state free precession cine images, T2-weighted imaging, and delayed gadolinium enhancement imaging. Additionally, free-breathing SENC images were obtained and then processed by a team of blinded cardiovascular imaging specialists using Myostrain software (Morrisville, USA). RESULTS/ANTICIPATED RESULTS: Left ventricular ejection fraction (LVEF) was normal in both groups (ï,³53%). Global longitudinal LV strain was significantly depressed in the ICI cardiotoxicity group versus controls (-12.8 ±3.2% vs. -16.6 ±1.9%, p=0.028). The average global circumferential LV strain was mildly abnormal (defined as strain > -17) in the ICI cardiotoxicity group and trended towards a higher value compared with controls (-16.0 ±2.6% vs -17.8 ±1.7%, p=0.103). The average number of dysfunctional segments (defined as strain > -10) was significantly higher in the ICI cardiotoxicity group (6.8 ±4.2 vs. 1.0 ±1.7, p=0.017). The proportion of abnormal myocardium was higher in the ICI cardiotoxicity group (66 ±21% vs. 45 ±18%, p=0.050), as well as the proportion of myocardium found to be dysfunctional (26 ±22% vs. 3.0 ±6.0%, p=0.041). DISCUSSION/SIGNIFICANCE OF FINDINGS: Despite having preserved LVEF, patients who met criteria for ICI-associated cardiotoxicity had both global and regional abnormal LV strain. Fast-SENC imaging may provide a sensitive tool for detection of early cardiotoxicity in this population. This study is limited by its small cohort and a larger prospective study would be of value.

